# P-699. The Impact of Low Influenza Immunization rates on U.S. Hospital System Resources. A Dynamic Model estimation

**DOI:** 10.1093/ofid/ofae631.895

**Published:** 2025-01-29

**Authors:** Van Nguyen, Joaquin F Mould-Quevedo

**Affiliations:** VHN Consulting, Montreal, Quebec, Canada; CSL Seqirus Inc., Summit, New Jersey

## Abstract

**Background:**

Increasing influenza vaccination rates can significantly reduce the onset of severe symptoms and reduce the risk of complications. This helps alleviate the burden during flu seasons when hospitals may be overwhelmed with patients. Nevertheless, the overall vaccine uptake has been decreasing in the United States which is expected to have an important effect by increasing the burden of disease. The aim of this study was to estimate the impact of low influenza vaccination rates on disease burden and U.S. hospital system resources.

**Methods:**

The impact of the reduced flu immunization rate was estimated using a dynamic age-stratified transmission model. Two U.S. flu seasons (2011-2012 for low incidence and 2017-2018 for high incidence) were used in the analysis to simulate the variation of flu epidemic. This study assessed four different flu vaccination rates: 25%, 30%, 35% and 40%. Outcome measures include number of infections, outpatient visits, hospitalizations, intense care unit (ICU) stays and deaths. The flu vaccine effectiveness (VE) rate was taken from CDC reports to estimate an average VE for all ages for the last 10 seasons (42%). Vaccination rates by age group were estimated using CDC reports and this model assumed immunization with quadrivalent flu vaccines for all ages. Total number of acute hospital and ICU hospital beds available for influenza were assumed in the U.S. at 300,000 and 30,000, respectively.

**Results:**

Using U.S. flu immunization rate from season 2023-2024 (≈35%), in case of a high flu incidence season, the total number of symptomatic infections is expected to result in 71M, office visits in 29M, hospitalizations in 0.94M and deaths in 133,670. Any scenarios with an immunization rate below 45% will generate significant pressure on the US hospital system and would saturate the number of ICU hospital beds in high incidence seasons. Only increasing the flu immunization rate up to 50% or higher may prevent any saturation of acute hospital or ICU hospital beds regardless on the incidence of the flu season.

**Conclusion:**

The U.S. analyses shows the critical need to increase the U.S. flu immunization rates to at least 50%, to improve health outcomes and avoid saturation of hospital system resources, especially those associated to ICU hospital beds.

**Disclosures:**

**Van Nguyen, PhD**, CSL Seqirus USA: Advisor/Consultant **Joaquin F. Mould-Quevedo, PhD**, CSL Seqirus USA: Stocks/Bonds (Private Company)

